# C-851-03. Burn Outcomes in Patients with Psychiatric Comorbidities: A National Database Study

**DOI:** 10.1093/jbcr/irag033.055

**Published:** 2026-04-06

**Authors:** Christopher J Fedor, Simon Rodriguez, Hilary Y Liu, Sarah M Tepe, Viktoria Voragen, Mare G Kaulakis, Mahi Pachgade, Jenny A Ziembicki, Francesco M Egro

**Affiliations:** University of Pittsburgh Medical Center Department of Plastic Surgery, Pittsburgh, PA; University of Pittsburgh School of Medicine, Pittsburgh, PA; University of Pittsburgh Medical Center Mercy Burn Center, Pittsburgh, PA; University of Pittsburgh Medical Center, Pittsburgh, PA; University of Pittsburgh School of Medicine, Pittsburgh, PA; University of Pittsburgh Medical Center Mercy Burn Center, Pittsburgh, PA; Drexel University College of Medicine, Plainsboro, New Jersey; University of Pittsburgh Medical Center, Pittsburgh, PA; University of Pittsburgh Medical Center, Pittsburgh, PA

## Abstract

**Introduction:**

Burn patients with psychiatric comorbidities are at higher risk for negative clinical course outcomes, such as extended hospital length of stay (LOS). However, the current literature is mainly based on single-site studies and shows conflicting results on its effects on outcomes, like mortality. This study presents the largest multicenter retrospective review to date, evaluating the impact of psychiatric comorbidities on burn patients while addressing inconsistencies in the existing literature.

**Methods:**

Using data from the Burn Care Quality Platform (BCQP) Registry from 2013 to 2022, a retrospective review was conducted on burn patients with psychiatric comorbidities. Variables extracted include patient demographics, burn characteristics, social factors, and clinical course data. Chi-square and multiple logistic regression models were used to identify potential risk factors, and descriptive statistics were used to summarize the data.

**Results:**

The study included 265 456 patients (57.8% male; mean age of 45.1 ± 17.5) were included in the study, of whom 23 936 (9.1%) had psychiatric comorbidities such as major psychiatric illness, major depressive disorder, and acute stress disorder. In patients with psychiatric comorbidities, commonly reported burn etiologies were flame burns (41.6% n = 9100), scald burns (20.5%, n = 4469), and contact burns (9.3%, n = 2041). Second-degree burns were most commonly reported (53.9%, n = 10 009), followed by third-degree (44.6%, n = 8372) and first-degree burns(1.5%, n = 278). Logistic regression models showed higher incidence of graft loss (OR:1.40, *p*≤.001), increased length of stay (OR:2.0, *p*≤.001), shorter ICU stays (OR:-0.89, *p*≤.001), and 72% increased mortality (OR:1.72 *p*≤.001) for patients with psychiatric comorbidities as compared to those without.

**Conclusions:**

Our research provides the most comprehensive retrospective analysis to date on the relationship between psychiatric comorbidities and burns. Patients with psychiatric diagnoses experienced poorer outcomes affecting both the patients and the consumption of hospital resources. This demonstrates the importance of humanistic support and proactive recognition of psychosocial needs. In addition, our conclusions help shed light on current discrepancies in the literature and reinforce the importance of acknowledging and addressing psychiatric comorbidities in burn patients to improve overall patient outcomes and safety.

**Applicability of Research to Practice:**

Our findings emphasize the need for psychiatric assessment in burn care protocols to better identify high-risk patients. Mental health considerations can guide personalized treatment strategies, improving overall patient outcomes.

**Funding for the study:**

N/A.

